# Using Time-Resolved Fluorescence to Measure Serum Venom-Specific IgE and IgG

**DOI:** 10.1371/journal.pone.0016741

**Published:** 2011-01-31

**Authors:** Pauline E. van Eeden, Michael D. Wiese, Susan Aulfrey, Belinda J. Hales, Shelley F. Stone, Simon G. A. Brown

**Affiliations:** 1 Centre for Clinical Research in Emergency Medicine, Western Australian Institute for Medical Research, University of Western Australia, Perth, Australia; 2 Samson Institute for Health Research, University of South Australia, Adelaide, Australia; 3 Department of Medicine, Royal Hobart Hospital, Hobart, Australia; 4 Centre for Child Health Research, Telethon Institute for Child Health Research, University of Western Australia, West Perth, Australia; 5 Department of Emergency Medicine, Royal Perth Hospital, Perth, Australia; University of Cincinnati College of Medicine, United States of America

## Abstract

We adapted DELFIA™ (dissociation-enhanced lanthanide fluoroimmunoassay), a time resolved fluorescence method, to quantitate whole venom specific and allergenic peptide-specific IgE (sIgE), sIgG_1_ and sIgG_4_ in serum from people clinically allergic to Australian native ant venoms, of which the predominant cause of allergy is jack jumper ant venom (JJAV). Intra-assay CV was 6.3% and inter-assay CV was 13.7% for JJAV sIgE. DELFIA and Phadia CAP JJAV sIgE results correlated well and had similar sensitivity and specificity for the detection of JJAV sIgE against intradermal skin testing as the gold standard. DELFIA was easily adapted for detecting sIgE to a panel of other native ant venoms.

## Introduction

The allergic response to insect stings can range from mild skin irritation through to large localised reactions and severe generalised (“systemic”) reactions which can result in fatal anaphylaxis. Insect venom allergy is a Type I or immediate hypersensitivity, where the allergenic peptide or protein cross-links venom-specific IgE (sIgE) bound to high-affinity IgE receptors (FcεRI) on mast cells and basophils, releasing preformed and newly-synthesized mediators [1]. Insect stings account for up to a third of anaphylaxis cases treated in Emergency Departments that serve suburban and rural populations and a quarter of all anaphylaxis deaths [2], [3].

Allergy to native ants of the genus *Myrmecia* is an important clinical problem in Australia. In the southern island state of Tasmania, 1% (95% CI 0.5–1.8) of the population has experienced one or more severe allergic reactions to a sting from *Myrmecia pilosula* (the “jack jumper ant” (JJA)) [4]. The efficacy of JJA venom immunotherapy (VIT) in preventing anaphylaxis has been demonstrated by a randomised double-blind, placebo controlled trial [5]. Three allergens from JJAV (Myr p 1, Myr p 2 and Myr p 3) have been identified and sequenced, including the only major allergen, the 5608 Da heterodimer Myr p 2 [6], [7], [8]. Myr p 2-specific IgE has been shown to bind to one of the chains in the heterodimer, designated Myr p 2a [8]. Allergic reactions to the sting of other *Myrmecia* species and other ant genera appear to be less common [4], [9], however the epidemiology is less well defined due in part to lack of sensitive and specific diagnostic tests for allergy to these species.

To diagnose specific-venom allergy, investigations to demonstrate the presence of sIgE are used to confirm the causative species and thus select an appropriate venom extract for VIT [10]. Methods include intradermal venom skin testing, quantitation of sIgE in serum and functional assays such as the histamine release test and flow-cytometry analysis of CD63 and CD203c upregulation on basophils [11], [12], [13], [14]. *In vitro* laboratory methods are thought to have lower sensitivity compared to intradermal venom skin testing [10]. For quantitation of sIgE, methods such as the Radio-Allergosorbent test (RAST) and Enzyme-Linked Immunosorbent Assay (ELISA) have evolved and been largely been replaced by the Phadia (previously Pharmacia) CAP® assay, which has greater sensitivity [15], and is highly automated and thus reliable and easy to run in high throughput clinical laboratories [16].

A recently introduced technique for highly sensitive detection and quantitation of sIgE against peanut and dust mite allergens is the DELFIA™ (dissociation-enhanced lanthanide fluoroimmunoassay, Wallac, Turku, Finland) which utilises a time resolved fluorescence signal [17], [18]. The advantages of this method include its log-fold dynamic range, low cost, flexibility for developing novel assays with new allergens in small numbers of patients, and that it does not require the use of hazardous materials such as radioactive iodine and cyanogen bromide.

We aimed to develop and validate a DELFIA assay for quantifying sIgE to JJAV and to the peptide allergen Myr p 2a, and then test whether this method was easily adaptable to detect sIgE to other native Australian ant venoms and to quantify sIgG_1_ and sIgG_4_ responses to venom immunotherapy.

## Methods

### Materials

Microtitre plates 96-well, Maxisorp Nunc, Roskilde, Denmark were obtained through In vitro Technologies Australia. Sodium bicarbonate (S6014), Trizma hydrochloride (T3253), Sodium chloride (S9888), Tween 20 (274348), sodium azide (S8032) and bovine serum albumin (A3059 & A9418) were purchased from Sigma-Aldrich, St Louis, Missouri. Anti-human IgE was purchased from Bioclone, Sydney, Australia and Becton Dickinson diagnostics, Australia. Europium-labelled streptavidin and enhancement solution Wallac, Oy were purchased through Perkin Elmer, Australia.

### Ethics Statement

Ethics clearances were provided by the Human Research Ethics Committee (Tasmanian) Network (H0008934) and the South Metropolitan Area Health Service Human Research Ethics Committee, Fremantle Hospital, Western Australia (05/258). All participants signed written consent forms. Ant nests were collected with the permission of all property owners. Ethics is not required for the collection of insects.

### Participants

Allergic sera and plasma samples were taken from participants with a history of systemic reactions to Australian native ant stings recruited through our venom allergy clinic at Royal Hobart Hospital or were enrolled in the Australian Ant Venom Allergy Study (AAVAS).

For participants with suspected allergy to JJA who presented to Royal Hobart Hospital, intradermal JJAV skin testing was performed with a positive skin test defined by wheal growth of at least 2 mm and flare greater than or equal to 10 mm in response to an intradermal injection of JJAV (about 0.02 ml to form a 3–5 mm bleb) at a concentration of 1.0 µg/ml or less. If an initial skin test was negative, the test was repeated 3–6 weeks later before declaring a negative result. JJA VIT was offered if the skin test was positive and further serum and plasma samples were taken immediately prior to commencing VIT then during VIT at approximately 1 week, 3 weeks, 2 months, 3 months, 6 months and 12–15 months.

Patients in the AAVAS study had reported a history of systemic reactions to one or more ant stings. Each patient sera was screened for sIgE to venom from ant species found in the state where the reaction occurred, and patients who had previous exposure to ant stings in other states of Australia were also screened for sIgE against species endemic to this/these states.

Negative control sera and plasma were collected from healthy volunteers in Western Australia who had recently arrived from overseas and had never been stung by an Australian ant. To assess the specificity of the assay in the presence of high total IgE, sera were also obtained from patients resident in the Northern Territory (where JJA are not endemic and thus exposure unlikely) with a variety of atopic conditions resulting in elevated IgE.

### Pooled Sera

To validate the assays, three different pooled sera were created. One, referred to as ‘standard sera’ from this point forward, was pooled from 21 JJAV allergic individuals with high levels of venom-specific IgE and used to create a standard curve. The second, referred to as ‘positive sera’ from this point forward, was created by pooling sera from a different cohort of 24 JJAV allergic individuals with quantifiable venom-specific IgE. The third consisted of pooled sera from JJA venom non-allergic individuals and was used as a negative control for all assays. Pooled sera were aliquoted and frozen at −80°C until required.

### Venom and Myr p 2a

JJAV was obtained from ants collected in Tasmania and analysed by previously reported methods to assure consistent allergen content prior to use [6], [19]. Myr p 2a was purchased from Proteomics International, Perth, Western Australia. Other ant venoms of clinical significance were extracted by venom sac dissection from ant nests collected into dry ice from all states of Australia. These were the large 15–30 mm long “bull dog” *Myrmecia* ants (*M forficata, M pyriformis, M gulosa, M gigas, M nigriceps, M brevinoda, M simillima, M tarsata,*), 10–15 mm long “jumper” *Myrmecia* ants similar in size and behaviour to the JJA (*M nigrocincta,*) and the small 6 mm “greenhead ant” (*Rhytidoponera metallica*). Prior to pooling venoms from different nests to form reference venoms for each species, worker ants from each nest were identified by an entomologist (Dr R W Taylor) and SDS-PAGE was performed to confirm identical band patterns.

### CAP

JJAV was coupled to CAP discs by Phadia and whole JJAV sIgE level was performed using the Phadia CAP® system (Uppsala, Sweden, recommended positive cut-off 0.35 IU/ml) [15] at SouthPath Laboratories, Bedford Park, South Australia.

### DELFIA for sIgE

Microtitre plates were coated with 100 µL of whole venom or synthetic Myr p 2a at 1, 2.5, 5, 10, 15 and 20 µg/mL in coating buffer (50 mM NaHCO_3_, pH 9.6) at 4°C overnight. After washing five times with wash buffer (50 mM Tris-HCl, 0.9% NaCl, 0.05% sodium azide, pH 7 with 0.01% Tween 20) plates were blocked overnight with bovine serum albumin (0.5% in wash buffer) at 4°C. After washing five times, 100 µL of sera was added in duplicate and incubated for 2 hours at 4°C. The plates were washed (X5) and incubated for 1 hour with 100 µL anti-human IgE (neat, 1/10, 1/20 and 1/100 in blocking buffer) at ambient temperature with shaking. Plates were washed (X5) then incubated for 30 minutes with 100 µL Europium-labelled streptavidin (1/1000 in blocking buffer), at ambient temperature with shaking. Following the final wash step, which consisted of eight washes, 100 µl enhancement solution at 22°C was added and incubated for 5 minutes before being read using the Wallac Victor 3 plate reader (Wallac, Oy). A positive control (positive sera) and negative control, were run in quadruplicate on every plate to assess reproducibility. The intra and inter-assay precision was determined by calculation of the mean, standard deviation and coefficient of variance of the results from positive sera on 50 assay plates. A titration of 2-fold serial dilutions of standard sera (1/2–1/128) was linear (R^2^>0.95) and used on every plate as an internal standard curve.

### IgE Standard Curve

Calibration of the standard curve was carried out by running the standard sera on the same plate as a set of Bioclone IgE standards (WHO standard 2nd IRP 75/502a calibrated). A polyclonal anti IgE antibody (Bioclone, Sydney, Australia) was used as the capture antibody as per the standard protocol - calibrators were 91.5, 45.75, 29.4, 9.37, 3.04 and 0 IU/ml. The limit of the blank (LoB), above which a positive result could be reported, was defined as mean plus 1.645 times the standard deviation of the negative control. The detection limit (LoD), at which a low level of sIgE should be reliably detected, was estimated as the mean plus two standard deviations of the assay negative control (Table 1). Our estimate of LoD assumed that the standard deviation for low positive samples would be the same as for negative controls. At the time we had not yet accumulated a suitable number of patient samples with low-level sIgE to verify this assumption.

**Table 1:** JJAV negative sera with high total IgE used to demonstrate assay specificity.

Sample No.	Total IgE (kUA/L)	Notes	CAP JJAVsIgE	DELFIA JJAV sIgE
08/75004	6,991	Recurrent pneumonia	NEG	NEG
07/262162	1,839	Probable rhinitis	NEG	NEG
08/41951	595	Chest infection	NEG	NEG
07/251692	493	Allergic rhinitis	NEG	NEG
08/99212	14,550	Crusted scabies	NEG	NEG
08/80397	19,700	Scabies protocol	NEG	NEG
07/232441	9,435	n/a	NEG	NEG
08/99214	624	Probable Allergic rhinitis	NEG	NEG
07/214495	584	Probable crusted scabies	NEG	NEG
08/099216	363	Rash/Cow's milk allergy	NEG	0.52
07/251690	3,012	Recurrent rhinosinusitis	NEG	NEG

### Applicability to Other Australian Ant Venoms

The LoB and LoD were calculated for the DELFIA sIgE assay using other ant venoms (*M forficata, M pyriformis, M gulosa, M gigas, M nigriceps, M brevinoda, M simillima, M tarsata, M nigrocincta, and R metallica*). For determination of sIgE against these venoms, plates were coated with 100 µl of 5 µg/ml venom and the method described above was followed. An IgE standard curve and positive and negative controls were included on each plate as described above.

### Applicability to JJAV and Myr p 2a specific IgG_1_ and IgG_4_

For the determination of JJAV and Myr p 2a specific IgG_1_ and IgG_4_, the method described above for IgE was followed, except that the anti-human IgE was replaced by anti-human IgG_1_ and IgG_4_ (Caltag, Invitrogen, Australia at a dilution of 1/750) and sera were diluted either 1/5 and 1/20 for IgG_1_ or 1/10 and 1/100 for IgG_4_. Two pools of sera high in JJA and Myr p 2a specific IgG_1_ and high in JJA and Myr p 2a specific IgG_4_ were prepared and used to create a standard curve, 1/20 to 1/1280 for IgG_1_ and 1/80 to 1/5120 for IgG_4_ and a positive control (1/20 on each plate). IgG_1_ and IgG_4_ standard curves were calibrated against a set of PeliClass human IgG subclass ELISA kit standards (WHO 67/97 reference preparation) (Cell Sciences, Canton, MA, USA.). All standard curves were linear. The pooled negative control was also included on each plate.

### Statistical Analysis

Sample means, standard deviations and variances were calculated using Microsoft Excel. Standard curves were prepared using linear regression in Microsoft Excel. Confidence intervals (binomial), Spearman correlations and graphs were prepared using Stata (Version 11) and GraphPad Prism (Version 5).

## Results

### Optimisation

The amount of venom and Myr p 2a peptide used for coating assay plates was determined by titration of the venom against a range of patient sera and found to be optimal at 5 µg/ml.

High background readings were initially a problem but this was reduced by selectively removing/reducing background causing reagents. For example in house wash buffer with and without Tween 20 was trialled against the commercial wash buffer with the in house buffer containing Tween 20 consistently showing lower background levels. Washing was found to be a crucial factor in the maintenance of low background levels, all washing steps consisted of 5 aspiration/wash cycles except for the final wash prior to the addition of the enhancement buffer which consisted of 8 aspiration/wash cycles. Various stages of the assay were found to be highly temperature sensitive. Therefore, initial plating out of sera was carried out with the plate on ice, while enhancement solution was stabilised at 22°C prior to its addition. To further reduce background readings a variety of sources of BSA were trialled with Fraction V, protease free and essentially gamma globulin free BSA chosen for routine preparation of blocking buffer. A phenomenon not so easily overcome or understood was the occurrence of so-called “hot wells”, the random appearance of an abnormally high count in a single well [20]. We have tried a variety of methods to overcome this effect, including centrifugation, filtration, temperature variation of sera and the enhancement buffer, as well as alternate buffers but, to date, nothing has eliminated the appearance of hot wells.

The anti-human IgE from Bioclone used in this assay was tested against the anti-human IgE antibodies from Becton Dickinson and found to be highly sensitive at lower concentrations of IgE and was thus chosen on this basis. The anti-human IgE antibody (Bioclone) was titrated and found to work equally well at 1/10 dilution and was thus used at this concentration in all experiments.

### Assay calibration

**Figure 1:**
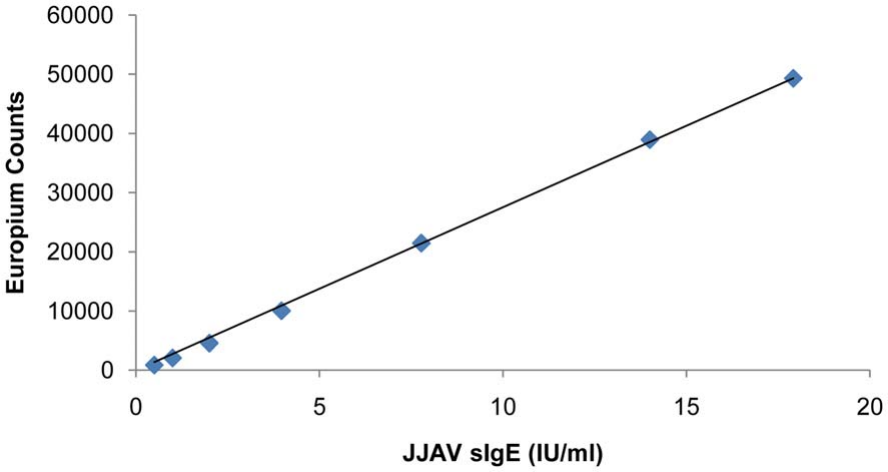
Typical standard curve of pooled positive JJAV IgE sera standards. Standards have been calibrated against Bioclone human IgE standard set to allow relative quantitation.

A typical JJAV sIgE pooled sera standard curve is shown in Figure 1.

### Specificity in presence of high total IgE

Characteristics of the patients (n = 11) whose sera were used to determine assay specificity is shown in Table 1. They were all found to be negative for JJAV sIgE by CAP however one was positive for JJAV sIgE by DELFIA. In addition, sera from 23 patients who suffered severe allergic reactions following clearly identified bulldog ant (BDA) stings in areas of Western Australia where JJA are very uncommon were all found to be negative for JJAV sIgE by DELFIA but positive to one or more BDA venoms.

### Assay Reproducibility

Low intra-assay variability was shown with an average coefficient of variance (CV) of 6.3% across 50 assays, and the inter-assay CV was 13.7% for JJAV sIgE.

### Quantitative correlation with CAP

**Figure 2:**
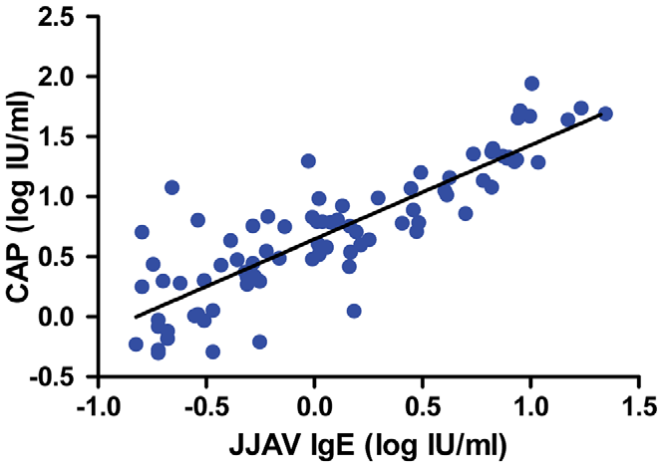
Correlation of CAP versus DELFIA assays for the determination of JJAV sIgE.

Both CAP and DELFIA tests were performed in 145 patients in whom 90 were positive using CAP, 84 were positive using DELFIA and 73 were positive using both methods. Where JJAV sIgE was detected by both assays there was a significant correlation between JJAV sIgE measured by CAP and DELFIA (p<0.001, Spearman's Rank Test) (Figure 2).

### Sensitivity and specificity with reference to intradermal venom skin testing in patients with a history of reactions to JJAV

A comparison of intradermal skin tests, CAP and DELFIA results is presented in Table 2. Sensitivity using DELFIA was 0.69 (95%CI 0.60–0.77) and for CAP was 0.74 (95%CI 0.65–0.81). Specificity for DELFIA was 0.96 (95%CI 0.78–1.0) and for CAP was 1.0 (95%CI 0.85–1.0).

**Table 2:** Sensitivity and specificity of CAP and DELFIA for detection of sIgE for JJAV with reference to intradermal venom skin testing.

		Intradermal venom skin test result	
		Positive	Negative
**CAP**	Positive	90	0
	Negative	32	23
**DELFIA**	Positive	84	1
	Negative	38	22

### Applicability to JJAV and Myr p 2 a specific IgG_1_ and IgG_4_

**Figure 3:**
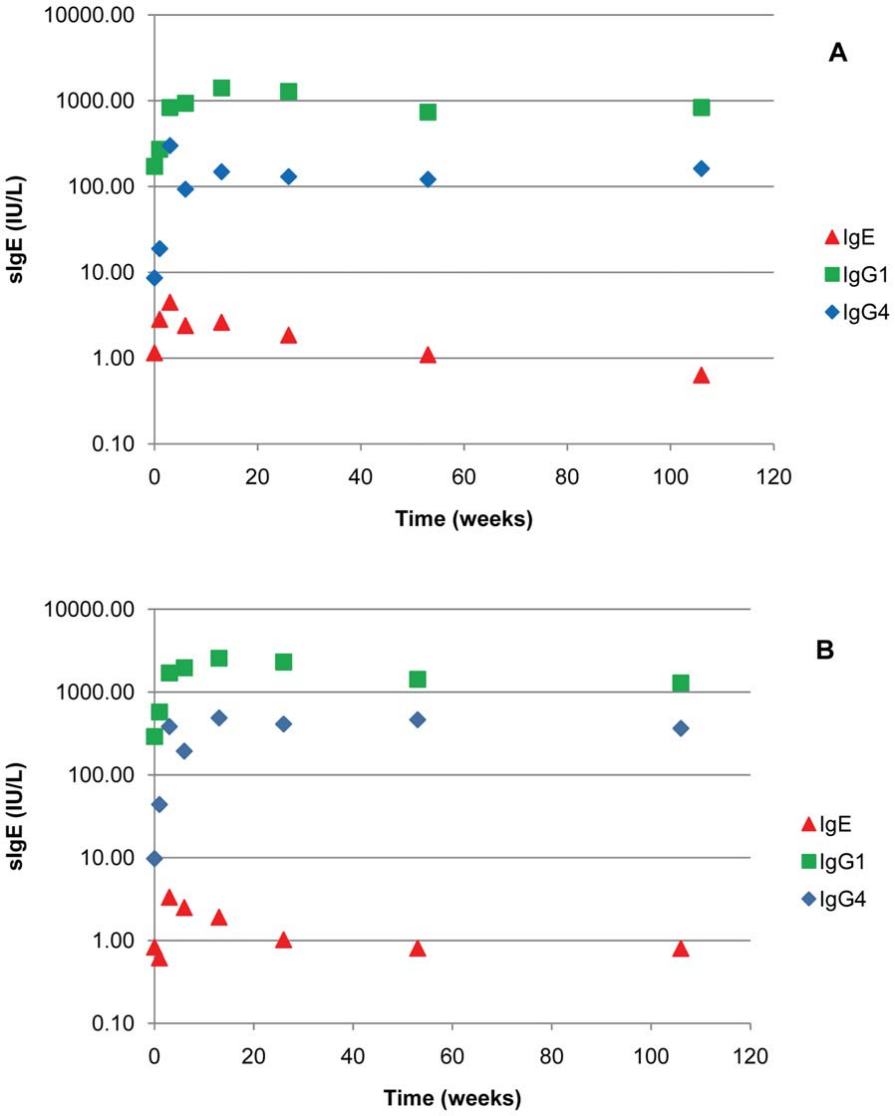
Specific IgE, IgG_1_ and IgG_4_ over a 2 year period from 10 patients undergoing VIT. A. Median values of JJAV and Myr p 2a. B. Specific antibody levels.

The LoB and LoD for sIgG_1_ and sIgG_4_ assays are presented in Table 3. Ten patients who underwent JJA VIT had a quantifiable amount of JJAV sIgE, sIgG_4_ and sIgG_1_ against JJAV prior to starting desensitisation. Figure 3 shows the levels of JJAV sIgE, sIgG_1_ and sIgG_4_ increased to a peak, then sIgE decreased to around initial levels, and IgG levels maintain a constant concentration.

**Table 3:** Calculated Limit of Blanks (LoB's) and Limit of Detection (LoD's) for JJAV and Myr p 2a specific IgG_1_ and IgG_4_.

	LoB (IU/ml)	LoD (IU/ml)
JJA IgE	0.15	0.18
Myr p 2a IgE	0.09	0.12
JJA IgG_1_	54.8	55.9
Myr p 2a IgG_1_	54.4	55.8
JJA IgG_4_	3.7	4.5
Myr p 2a IgG_4_	6.4	8.2

### Applicability to other Myrmecia Venoms

The DELFIA has also been adapted for use with other *Myrmecia* venoms. The number of sera screened, number of positives, LoB, LoD, lower, median, and upper quartile range for positives for each venom are shown in Table 4. The sensitivity and specificity of these assays could not be determined for these species because appropriately standardised venom preparations for intradermal venom skin testing in humans were not available.

**Table 4:** Calculated LoBs and LoDs for other ant venom sIgE.

Species	LoB (IU/ml)	LoD (IU/ml)	No of Patients screened	No of positives	Lower Quartile (IU/ml)	Median (IU/ml)	Upper Quartile (IU/ml)
*M. forficata*	0.02	0.13	272	44	0.22	0.42	0.94
*M. pyriformis*	0.04	0.09	284	40	0.18	0.37	1.41
*M. gulosa*	0.01	0.04	130	7	0.34	0.39	0.71
*M. gigas*	0.02	0.06	130	7	0.09	0.14	0.17
*M. nigriceps*	0.02	0.11	251	51	0.19	0.36	0.96
*M. brevinoda*	0.03	0.09	131	7	0.18	0.19	0.22
*M. simillima*	0.04	0.05	151	37	0.09	0.11	0.19
*M. tarsata*	0.08	0.1	151	13	0.25	0.37	0.66
*M. nigrocincta*	0.01	0.12	131	18	0.28	0.56	3.13
*R. metallica*	0.12	0.28	128	42	0.63	1.71	4.25

## Discussion

We have developed a time-resolved fluorometric assay (DELFIA) to detect JJAV sIgE, and the assay has been adapted to be used with other native ant venoms (*Myrmecia* and *Rhytidoponera* species), in addition to specific venom allergens (Myr p 2a) and for the detection of JJAV specific IgG_4_ and IgG_1_. When using venom skin testing as a diagnostic reference, sensitivities and specificities of DELFIA were equivalent to Phadia CAP for JJAV sIgE.

The main strengths of this method are its flexibility, log-fold dynamic range, cost effectiveness and potential for high-throughput screening. This is particularly useful for dealing with novel allergens and in the research setting, where detection of allergen specific IgG subclass levels may be useful. The assay has a convenient and rapid protocol. Plates can be coated up to a week in advance and stored at 4°C allowing batch preparation. This results in reduced wastage of reagents, and once the plates have been set up the assay is complete in 4.5 hours. However, limitations of this technique include the sensitivity of the assay to subtle temperature changes and the large number of wash cycles that are required, although this limitation can be overcome with automation. The “hot well’ phenomenon is a continuing problem being the cause of many repeats [20], however, this was not the case of more stable purified antigens such as peanut allergens[17] or House Dust Mite allergens[18].

Intradermal venom skin testing is currently used as the gold standard for venom allergy diagnosis as it is more sensitive for low levels of sIgE, which is concentrated on the surface of skin mast cells. The DELFIA and CAP were comparable for sensitivity however some patients positive to one assay were negative to the other and visa versa. This is probably because at low levels of sIgE (approaching the LoB) these assays may not always reliably detect allergen, introducing a degree of chance as to whether sIgE is detected. Likewise in some settings a skin test may be negative and a serum test may be positive so that the occasional case showing detectable serum sIgE but a negative skin test will occur (Table 2). One patient with high levels of total IgE used here (Table 1) to test specificity, showed a positive result in the DELFIA assay. However none of these patients have been skin tested for JJAV and it is possible that this patient had previous exposure while resident or visiting areas of Australia where the JJA is prevalent.

A number of patients were noted to have a higher sIgE value for Myr p 2a compared to JJAV. The plates used in the DELFIA assay bind venom peptides and proteins to a polystyrene solid phase via non-covalent interactions. Given the unique charge state of each venom peptide in the binding buffer, each will have a different affinity for the solid phase, and therefore the venom peptides that are bound to the solid phase may not be quantitatively representative of whole venom. Although Myr p 2a comprises approximately half of the protein content of JJA venom [6], it is highly basic and may have a lower affinity for the solid phase than other venom components, which may result in a lower proportion of it binding to the solid phase compared to what is present in whole venom. Therefore, sera that recognise the Myr p 2a allergen may have a significantly lower DELFIA result when whole JJA venom is bound to the solid phase compared to Myr p 2a. This is less likely to be the case with CAP assays, where each CAP has a higher allergen binding capacity [15]. This higher protein binding capacity translates to a greater venom specific IgE binding capacity, and may account for the observation that, on average, the CAP assay determined there was more venom sIgE present compared to the DELFIA assay. These are inherent weaknesses to any method that utilises such solid phases with lower protein binding capacity to bind complex allergen mixtures.

For these reasons, DELFIA is best considered a semi-quantitative assay when being used to compare the relative amount of venom specific IgE, IgG_1_ and IgG_4_ between individuals or changes in individual patients over time, but in the context of diagnosing the presence of IgE antibodies, it produces similar results compared to CAP with respect to sensitivity and selectivity.

The DELFIA has proved a useful tool for measuring JJAV sIgE in patient sera and plasma and it is easily adaptable to be used to detect IgE specific for venom peptide allergens and other ant venoms and for measuring venom specific IgG_1_ and IgG_4_.
